# Crosstalk Defect Detection Method Based on Salient Color Channel Frequency Domain Filtering

**DOI:** 10.3390/s22145426

**Published:** 2022-07-20

**Authors:** Wenqiang Xie, Huaixin Chen, Zhixi Wang, Xing Liu, Biyuan Liu, Lingyu Shuai

**Affiliations:** 1 Department of Resources and Environment, University of Electronic Science and Technology of China, Chengdu 611731, China; wqxie@std.uestc.edu.cn (W.X.); wangzx.rd@trulyopto.cn (Z.W.); mrliuxing@std.uestc.edu.cn (X.L.); byliu@std.uestc.edu.cn (B.L.); lyshuai@std.uestc.edu.cn (L.S.); 2 Novel Product R & D Department, Truly Opto-Electronics Co., Ltd., Shanwei 516600, China

**Keywords:** liquid crystal display, defect detection, crosstalk defect, salient color channel, frequency-domain saliency

## Abstract

Display crosstalk defect detection is an important link in the display quality inspection process. We propose a crosstalk defect detection method based on salient color channel frequency domain filtering. Firstly, the salient color channel in RGBY is selected by the maximum relative entropy criterion, and the color quaternion matrix of the displayed image is formed with the Lab color space. Secondly, the image color quaternion matrix is converted into the logarithmic spectrum in the frequency domain through the hyper-complex Fourier transform. Finally, Gaussian threshold band-pass filtering and hyper-complex inverse Fourier transform are used to separate the low-contrast defects and background of the display image. The experimental results show that the accuracy of the proposed algorithm reaches 96% for a variety of crosstalk defect detection. Compared with the current advanced defect detection algorithms, the effectiveness of the proposed method for low-contrast crosstalk defect detection is confirmed.

## 1. Introduction

Display quality inspection plays a key role in the display production process. The existing display quality detection process still relies on manual detection, which is affected by the subjective feelings of the inspectors along with the problems of low efficiency and unstable accuracy [1]. Particularly on small wearable devices, the defect is imperceptible to the human eye when it is close to the background. Therefore, the use of machine vision and digital image processing technology for display defect detection has become an urgent problem to be solved.

The existing display defect detection methods are mainly divided into three types: methods based on image registration [2,3,4], background reconstruction [5,6,7,8,9,10], and deep learning [11,12,13,14,15,16,17]. Shuai et al. [2] proposed the method of histogram equalization to adjust the brightness of the registered image, which can effectively suppress the problem of edge afterimages caused by the unaligned edges, and extract multiscale defects. However, this method didn’t perform image registration on a solid-color background. Yang et al. [6] proposed a method based on abnormal region detection and level set segmentation for Mura defects. They analyzed the shortcomings of polynomial fitting, used polynomial fitting to obtain candidate abnormal regions, and then applied the level set method for accurate defect segmentation. This method can detect weak contrast defects, but is unable to obtain defect-free areas due to the large defect area, and it fails to detect abnormal areas or overcome edge problems. Zhu et al. [11] proposed a hierarchical multi-frequency-based channel attention network, which utilizes the attention mechanism to weight scratch defects with different aspect ratios, effectively realizing the detection of defects of different shapes. Zhu et al. [12] proposed to use the Yolov3 [18] for detecting point-like and abnormally displayed defects, which can effectively detect defects in multiple backgrounds simultaneously. Chang et al. [13] proposed a method combining image preprocessing and the convolutional neural network (CNN), and designed a strategy to solve the problem of sample imbalance due to small defect areas and large backgrounds in the image. The deep learning method requires the construction of a large data set to guarantee the detection capability, and thus fails to deal with small sample data effectively. Lo et al. [17] proposed to use the Zernike moments to extract image features and elliptic basis function neural networks for classification, which can effectively detect multiple defects.

The crosstalk picture is a specific display pattern during the display quality detection process, which is designed for testing the crosstalk display capability. A crosstalk picture that cannot be displayed properly by a defective display screen is called a crosstalk defect. There are two main situations where the detection of crosstalk defects is especially difficult: (1) when the defect covers a large area of nearby half of the screen—it appears as continuous color lines with variable colors; (2) when the contrast between the defect area and the background is not fixed—low contrast makes it difficult to detect the defect area.

The existing methods present some difficulties in the detection of crosstalk defects, so we propose a crosstalk defect detection method based on salient color channel and frequency domain filtering. For the problem of a large crosstalk defect area, we adopted the salient color feature extraction method [19], used relative entropy to adaptively select the salient color channel, and combined it with the Lab color space [20] to form the image color quaternion matrix [21]. For the problem of low contrast in defect areas, we adopted the frequency-domain Gaussian threshold screening and band-pass filtering (GTB) method, which significantly enhances the saliency of low-contrast defects.

Our contributions are as follows:

(1) A new crosstalk defect detection method is proposed, which combines color feature extraction and frequency-domain GTB filtering to achieve efficient and accurate detection of crosstalk defects under low contrast and strong background noise.
(2) An adaptive salient color channel selection method is proposed, which can retain salient color features for large defects and solve the problem of difficult feature extraction.
(3) The GTB frequency-domain filtering method is proposed, which enhances the salient regions of defects and suppresses the interference of background noise, and realizes the effective separation of low-contrast crosstalk defects and background noise.

This article is organized as follows: Section 2 presents related work, Section 3 describes the proposed color saliency channel selection method and frequency-domain GTB filtering method, Section 4 discusses the experimental results, and Section 5 summarizes the content of this paper.

## 2. Related Works

In the past ten years, with the rapid development of display technology, a large number of methods for detecting weak contrast defects in display screens have appeared. Ngo et al. [5] used low-pass filtering on the input image, polynomial fitting, and discrete cosine transform to reconstruct the background, obtained multiple defect shadow maps, and used threshold segmentation for defect detection. Jin et al. [22] proposed a method for Mura defect detection using discrete cosine transform (DCT) background reconstruction and bi-segment exponential transform. The bi-segment exponential transform effectively enhances the contrast of low-contrast defects, and the Otsu’s method is used to achieve accurate segmentation of defects. Fan et al. [23] used polynomial fitting for background reconstruction and threshold segmentation to obtain defect candidate regions, which could efficiently detect low-contrast defects. Cui et al. [24] adopted the Otsu’s method to select defect candidate regions, then used variance and meshing to detect Mura and edge defects. In addition to the above methods, there are defect detection methods based on defect features, such as color feature [25], similarity of histogram [26], and the dictionary learning method [27].

The saliency target detection method is mainly aimed at the target area that the human eye is most interested in natural scenes. Itti et al. [19] proposed to fuse objects by color feature, brightness feature, and scale feature to obtain saliency object regions. Guo et al. [28] proposed to use a combination of color features and motion features to highlight salient regions using the phase spectrum in the frequency domain. Li et al. [29] proposed to use of a Gaussian function to convolve the amplitude spectrum in the frequency domain, which can effectively highlight the salient target area, and the parameters of the Gaussian function are determined by the scale of the salient target. The saliency method can effectively obtain the human eye’s perceptual area, and some saliency-based methods have been used for defect detection [30,31,32]. Liu et al. [33] improved the hyper-complex Fourier transform (HFT) method by adding two-dimensional entropy features to the input features, which achieved effective extraction of fabric defects.

## 3. Methodology

### 3.1. Algorithm Architecture

**Figure 1:**
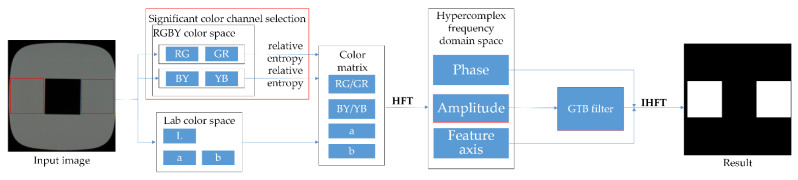
Algorithm Architecture.

Figure 1 shows the framework of the crosstalk defect detection method based on significant color channel frequency domain filtering.

Firstly, the original image is converted between the RGBY color space and the Lab color space, and the relative entropy maximum criterion is used for the opposite color channels in the RGBY space to evaluate the saliency of channel defects. Then the salient color channel and Lab color space are selected to form the image color quaternion matrix. Secondly, the quaternion color matrix is transformed into a hyper-complex frequency domain space using the HFT, while the magnitude spectrum is processed using GTB filtering. Finally, the inverse hyper-complex Fourier transform (IHFT) is employed to obtain the frequency domain saliency map, which then undergoes region segmentation to obtain the defect detection result.

### 3.2. Salient Color Channel Selection

The RGBY color space [19] adopts the human visual competition mechanism, which hinders the effective selection of the color channel with better defect protrusion and background suppression when detecting crosstalk defects. The background information and target information contained in different channels are varied, so we design a competitive mechanism between defective targets and backgrounds for feature extraction, which retains as many targets as possible while suppressing the background.

The human visual competition color space RGBY proposed by [19] is generally used in saliency detection.

It is calculated as:

(1) $$\{\begin{array}{c} R=r-\frac{g+b}{2},\\ G=g-\frac{r+b}{2},\\ B=b-\frac{r+g}{2},\\ Y=\frac{r+g}{2}-\frac{\left|r-g\right|}{2}-b, \end{array}$$

(2) $$\{\begin{array}{c} RG=R-G,\\ BY=B-Y,\\ I=\frac{r+g+b}{3}, \end{array}.\;$$

where *r*, *g* and *b* are the three channels of the original image. The RGBY color space decomposes the *RGB* input image into two parts: the RGBY color feature and the luminance (*I*). It is pointed out by [34] that human vision is used for the color competition mechanism, and the RGBY competition color space is generated. The RGBY color feature implements the aberration operation in this paper, which can roughly suppress the background and highlight the abnormal part.

Based on the original *RG* and *BY* channels, the opposite space *GR* and *YB* channels are added, which are expressed as:

(3) $$\{\begin{array}{c} GR=G-R,\\ YB=Y-B, \end{array}$$

By comparing the area difference of *RG*, *GR*, and *BY*, *YB* between the defect and the background, the opposite color channel is adaptively selected. Since it is difficult to directly calculate the area of the defect, we use the calculation of the area of the background to achieve this. When the background area contained in the opposite space is large, we should discard this channel. The comparison of the area part adopts the relative entropy calculation, and the calculation formula of the relative entropy:

(4) $$H_{kl}\left(P∥Q\right)=P\left(x\right)log\sum^{}\frac{P\left(x\right)}{Q\left(x\right)},\;$$

The relative entropy represents the difference between the grayscale distribution P(*x*) of the input feature P and the grayscale distribution Q(*x*) of the input feature *Q*. When the two are the same, $H_{kl}\;$= 0, it can effectively represent the feature’s distribution distance. The brightness channel contains stable background information, so we calculate the relative entropy between the RGBY color channel and the *I* brightness respectively, and retain the opposite feature space with a large value.

The maximum entropy criterion is described as:

(5) $$\{\begin{array}{c} H_{kl}\left(RG,I\right)>H_{kl}\left(GR,I\right),f_{1}=RG,\\ H_{kl}\left(RG,I\right)\leq H_{kl}\left(GR,I\right),f_{1}=GR, \end{array}$$

(6) $$\{\begin{array}{c} H_{kl}\left(BY,I\right)>H_{kl}\left(YB,I\right),f_{2}=BY,\\ H_{kl}\left(BY,I\right)\leq H_{kl}\left(YB,I\right),f_{2}=YB, \end{array}$$

### 3.3. Frequency Domain GTB Filtering

#### 3.3.1. Quaternion Representation and Hypercomplex Fourier Transform

The Lab color space conforms to the perceptual properties of the human eye [20], which preserves sufficient colors within the corresponding color channels. The color channel represented by the ab color channel is relatively consistent with the description of the RGBY opposite space, so the ab color channel and the RGBY color feature are used to form the quaternion color feature matrix.

The image quaternion color matrix is represented as follows:

(7) $$f\left(n,m\right)=f_{1}+f_{2}i+f_{3}j+f_{4}k,$$

where i,  j and k represent the imaginary axes that satisfy $i^{2}=j^{2}=k^{2}=ijk=-1$. $f_{3}$. is the a channel of the Lab color space and $f_{4}$ is the b channel of the Lab space.

The image quaternion matrix is transformed to the frequency domain space using the hyper-complex Fourier transform, calculated as follows:

(8) $$F_{H}\left[u,v\right]=\frac{1}{\sqrt{MN}}\overset{M-1}{\underset{m=0}{\sum }}\overset{N-1}{\underset{n=0}{\sum }}e^{\mu 2\pi \left(\left(\frac{mv}{M}\right)+\left(\frac{mu}{N}\right)\right)}f\left(n,m\right),$$

where μ. is a quaternion unit, $\mu^{2}=-1$.

Inverse transform of the hyper-complex Fourier transform is calculated as follows:

(9) $$f_{h}\left(n,m\right)=\frac{1}{\sqrt{MN}}\overset{M-1}{\underset{v=0}{\sum }}\overset{N-1}{\underset{u=0}{\sum }}e^{\mu 2\pi \left(\left(\frac{mv}{M}\right)+\left(\frac{mu}{N}\right)\right)}F_{H}\left(u,v\right).$$

The amplitude spectrum, phase spectrum, and Eigen-axis spectrum are calculated as follows:

(10) $$Am\left(u,v\right)=\left|F_{H}\left[u,v\right]\right|,$$

(11) $$P\left(u,v\right)=tan^{-1}\frac{Img\left(F_{H}\left[u,v\right]\right)}{Real\left(F_{H}\left[u,v\right]\right)},$$

(12) $$\chi \left(u,v\right)=\frac{Img\left(F_{H}\left[u,v\right]\right)}{\left|Img\left(F_{H}\left[u,v\right]\right)\right|},$$

where |·| is the modulo operation; Img is imaginary part computation; Real is real part computation; Am(u,v) is the magnitude spectrum; P(u,v) is the phase spectrum; χ(u,v) is a pure quaternion matrix.

To compute the magnitude spectrum of an image quaternion matrix, it can be converted to a log spectrum as follows:

(13) A(u,v)=log(Am(u,v)+1),

#### 3.3.2. Gaussian Filter Parameter Optimization

To enhance the saliency of the defect region, the magnitude spectrum is convolved with a Gaussian template. The existing way to obtain saliency results is to perform an information entropy calculation on the obtained saliency map, and the goal of maximum entropy is the required saliency map. Through experimental analysis, the best saliency map of crosstalk defects can be obtained by calculating the minimum entropy of the magnitude spectrum.

Define the size of the input image as (M,N). Set the range of template dimensions k and σ to:

(14) $$k_{n}=\min\left\{M,N\right\}\times 0.01\times n,\left(n=1,2,3,\ldots ,8\right),$$

(15) $$\sigma_{n}=3^{n-2},\left(n=1,2,3,\ldots ,8\right).$$

The high-contrast crosstalk defect image is selected for the optimal Gaussian template parameter selection, to illustrate that the extraction of information entropy from the frequency domain amplitude spectrum can replace the information entropy extraction of the saliency map. The saliency map obtained by different Gaussian templates, for example, is evaluated using the saliency indicator NSS (Normalized Scanpath Saliency, NSS) [35] to determine the effectiveness of the optimal parameters.

(16) $$\mathrm{NSS}\left(P,Q^{B}\right)=\frac{1}{N}\underset{i}{\sum }\bar{P_{i}}\times Q_{i}^{B},$$

(17) $$N=\underset{i}{\sum }Q_{i}^{B},$$

(18) $$\bar{P}=\frac{P-\mu \left(P\right)}{\sigma \left(P\right)},$$

where P denotes the saliency map of the input. $Q_{i}^{B}$ is the binary map of the target area of the input saliency map. The one-dimensional entropy is calculated as follow:

(19) $$H=-\sum^{}\left(p\times \right),$$

where H represents the information entropy of the amplitude spectrum and p represents the statistics of the gray histogram of the amplitude spectrum.

**Figure 2:**
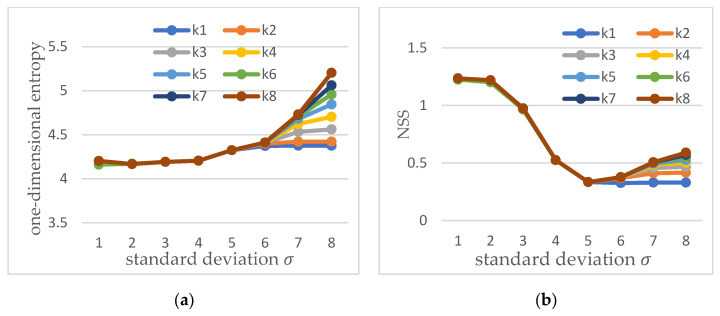
(**a**) Entropy value obtained by Gaussian parameter template, (**b**) saliency evaluation result.

We use different Gaussian functions to perform Gaussian convolution on the original amplitude spectrum, use one-dimensional entropy to calculate the entropy value of the original image, and use NSS to evaluate all the saliency maps obtained after convolution, as shown in Figure 2.

The horizontal axes in Figure 2 represent the parameters of the standard deviation of different Gaussian templates. The vertical axis in Figure 2a is the one-dimensional entropy value of the amplitude spectrum, and the vertical axis in Figure 2b is the NSS value of the saliency map. The results of data analysis show that the minimum entropy value can obtain the best saliency map of crosstalk defects. Figure 2 also shows that the size of the Gaussian window has little effect on the saliency map, and only affects the generation of the saliency map when the standard deviation is large.

#### 3.3.3. Frequency Domain Threshold Screening and Bandpass Filtering

To enhance the saliency of the defect area and suppress the background noise, threshold screening and band-pass filtering are performed on the magnitude spectrum after Gaussian convolution.

Threshold screening is needed to calculate the mean and standard deviation of the original amplitude spectrum. The calculation formula is as follows:

(20) $$th=\mu s_{max}+K\delta s_{min},$$

(21) $$\mu s=\frac{1}{M\times N}\overset{M}{\underset{i=1}{\sum }}\overset{N}{\underset{j=1}{\sum }}A{\left(u,v\right)}_{i,j},$$

(22) $$\delta s=\sqrt{\frac{1}{M\times N}\overset{M}{\underset{i=1}{\sum }}\overset{N}{\underset{j=1}{\sum }}{\left(A\left(u,v\right)-\mu \right)}^{2}}.$$

where th is the amplitude spectrum segmentation threshold; A(u,v) is the original logarithmic amplitude spectrum; μs is the mean value of the original logarithmic amplitude spectrum; δs is the standard deviation of the original logarithmic amplitude spectrum. K is selected according to the actual image. Following Gaussian convolution, threshold filtering is performed on the amplitude spectrum, amplitude values in the amplitude spectrum that are greater than the threshold value are retained, and the amplitude spectrum after threshold filtering can be obtained.

The filtering conditions are as follows:

(23) $$\{\begin{array}{c} F\left(w\right),F\left(w\right)\geq th,\\ 0,\;\;else, \end{array}$$

where F(w) is the amplitude value after Gaussian convolution.

The selection of the band-pass requires a comparison of the amplitude spectrum of the defect-free area and the threshold-screened amplitude spectrum of the defect.

A suitable band-pass filter needs to be designed to filter the amplitude spectrum filtered by the threshold. The filter is described as:

(24) $$H\left(u,v\right)=\{\begin{array}{c} 1,D_{0}+\frac{W}{2}\geq D\left(u,v\right)\geq D_{0}-\frac{W}{2}\\ 0,\;\;\;\;\;\;\;\;else. \end{array},$$

where H(u,v) is a band-pass filter, and the pass-band range is $\left(D_{0}+\frac{W}{2},D_{0}-\frac{W}{2}\right)$ and its range is determined by the actual situation. The defect amplitude spectral information is obtained after using band-pass filtering. An inverse hyper-complex Fourier transform is performed on the defect magnitude spectrum:

(25) $$S=F_{H}^{-1}\left\{\exp(F{\left(w\right)}^{\prime }{}_{\;}-1)P\left(u,v\right)\chi \left(u,v\right)\right\},$$

where $F{\left(w\right)}^{\prime }$ represents the amplitude spectrum retained after bandpass filtering, S represents the obtained defect saliency map, P(u ,v) is the original phase, and χ(u ,v) is the characteristic axis spectrum.

## 4. Experimental Results

### 4.1. Crosstalk Defect Data and Image Quality Evaluation

**Figure 3:**
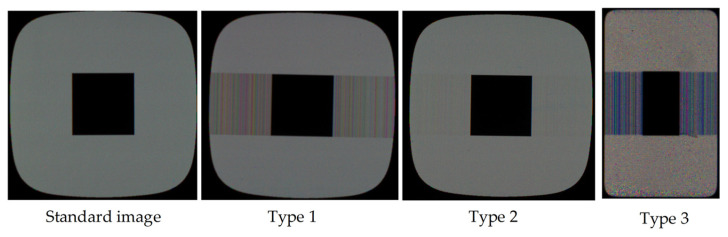
Different types of input images.

The three main types of crosstalk defect are shown in Figure 3. In Type 1, the defected part has high contrast and there is low speckle noise in the background; Type 2 has low contrast between the gray level of the defect and the background, and there is less speckle noise in the background; in Type 3, the defected part has high contrast, and the background contains a lot of noise. In the following discussion, we use Type 1 for high-contrast, low-noise defect maps, Type 2 for low-contrast, low-noise defect maps, and Type 3 for high-contrast, high-noise defect maps. All compared display defect detection methods were programmed using MATLABR2018b and all experiments were performed on the same computer with Intel Core i7-7700 CPU@3.60 GHz, 16 GB RAM, and Windows 7 64-bit operation system.

To quantitatively evaluate the relationship between defects and background in the original image, PSNR (Peak Signal to Noise Ratio) [36] and MSE (Mean Square Error, MSE) [37] metrics are used.

(26) $$\mathrm{PSNR}=10\log_{10}\frac{\left(2^{n}-1\right)}{MSE},$$

(27) $$\mathrm{MSE}=\left|std_{b}-std_{t}\right|,$$

where $std_{b}$ is the standard deviation of the background, and $std_{t}$ is the standard deviation of the defect.

The image quality evaluation results are shown in Table 1:

The value of MSE in Table 1 changes more in the three types of images, and the value of PSNR changes less. Among them, Type 3 has the largest background noise, so its MSE value is also the largest. In Type 2, the contrast and noise of background and defects are both low, so its MSE value is small but the PSNR value is the largest. The MSE and PSNR values in Type 1 are in an intermediate state compared to the other two types.

### 4.2. Color Channel Significance Analysis

In HFT [27] and PQFT [26], the input image is transformed into a quaternion space composed of various features, and then the frequency domain saliency analysis is performed using the hyper-complex Fourier transform. We analyzed the effect of several commonly used features on crosstalk defects and finally concluded that only color features can effectively represent the features of crosstalk defects; using other types of defect information results in defects remaining unextractable. The features analyzed in this paper are color feature space, two-dimensional information entropy feature, and brightness feature. Color feature spaces include RGBY color space, Lab color space, and HSV color space.

**Figure 4:**
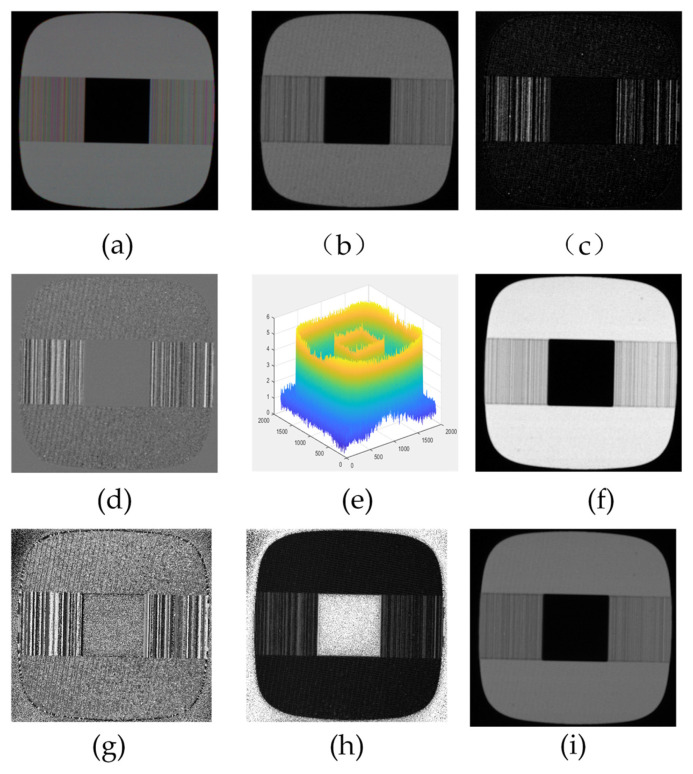
Decomposed feature map of various features of the original image. (**a**) Original image, (**b**) RGB space R channel, (**c**) RGBY space R channel, (**d**) Lab space a channel, (**e**) Two-dimensional information entropy H feature, (**f**) Average luminance feature *I*, (**g**) HSV spatial H channel, (**h**) HSV spatial S channel, (**i**) HSV space V channel.

As shown in Figure 4, after the feature decomposition of the original image, the average brightness feature in RGB space and the V channel in HSV space are consistent with the original image, without suppressing the background or enhancing the defects. However, the two-dimensional information entropy feature does not describe the defect feature well, which over-enhances the edge information and drowns the defect information. It can be seen that the more effective defect feature descriptions are mainly in the RGBY space and the ab channel of the Lab color channel, as well as the H and S channels of the HSV space.

We use both SCRG (Signal-to-clutter Ratio Gain) and BSF (Background Suppression Factor) [38] to calculate the performance of the feature space.

(28) $$\mathrm{SCRG}=\frac{{\mathrm{SCR}}_{out}}{{\mathrm{SCR}}_{in}},$$

(29) $$\mathrm{SCR}=\frac{\left|\mu_{T}-\mu_{B}\right|}{\sigma_{B}},$$

(30) $$\mathrm{BSF}=\frac{\sigma_{Bin}}{\sigma_{Bout}},$$

${\mathrm{SCR}}_{in}$ and ${\mathrm{SCR}}_{out}$ represent the image signal-to-clutter ratios (SCRs) of the input image and the modulo image. $\mu_{T}$ is the gray mean of the defect area, and the mean and standard deviation of the target neighborhood of $\mu_{B}$. and $\sigma_{B}$. The signal-to-noise ratio gain represents the signal-to-noise ratio of the output feature map in the feature space, and the background suppression factor represents the degree of difference between the defect and the background.

We use SCRG and BSF in RGB space as benchmarks for comparison. When the above two parameters are close to the effect of RGB space, this type of feature can’t effectively separate background and defect information.

As shown in Table 2, the BSF parameter value of HSV space is the largest, but its SCRG parameter is close to the value of RGB space, so it cannot effectively separate defect information. The Lab color space and the RGBY color feature can deviate effectively from the RGB space in terms of the two parameters of SCRG and BSF, so we choose these two color spaces as the input features to construct the color quaternion matrix.

### 4.3. GTB Experiment Comparison and Result Analysis

To illustrate the effectiveness of using the GTB approach, we compare the saliency maps obtained using GTB with only Gaussian template convolution.

**Figure 5:**
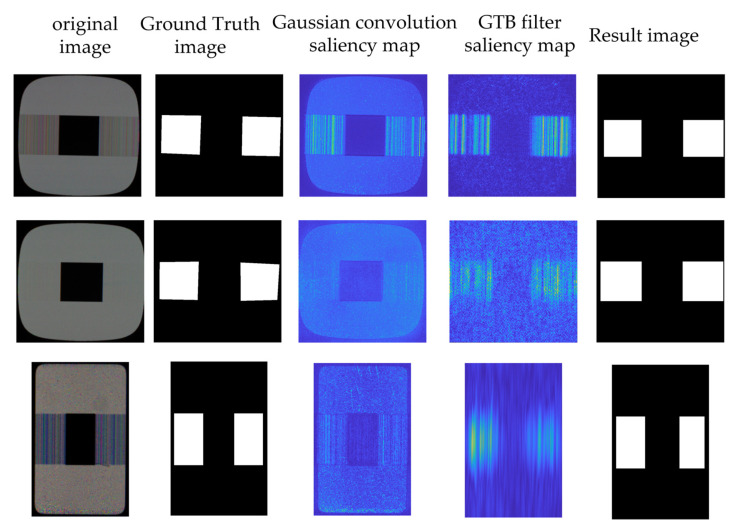
Comparison of GTB methods.

**Figure 6:**
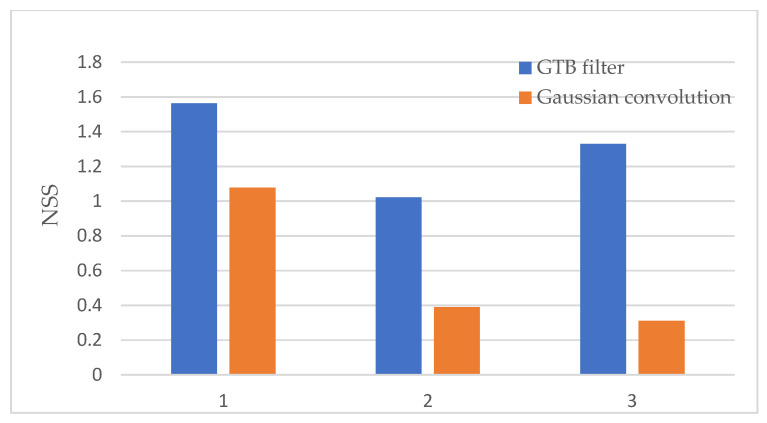
NSS value results for GTB method and Gaussian method.

As shown in Figure 5 and Figure 6, only the Gaussian convolution method can significantly enhance the crosstalk defect with strong contrast, while the saliency calculation method of the Gaussian convolution cannot suppress the point-like noise, and thus cannot effectively separate the defect and the background. After using the GTB method, the defect information is further enhanced, and weak contrast defects can be effectively detected. The NSS indicator of the saliency map shows that the saliency for Type 1 is improved by 45%, the saliency of Type 2 is increased by 162%, and the saliency of Type 3 is increased by 327%, thus demonstrating that our method can detect faults more effectively.

We evaluated the detection performance of our algorithm. Two evaluation metrics are used: TDR and FDR. TDR is defined as the sum of correctly detected pixels in the test image divided by the sum of true crosstalk defect pixels, and FDR is defined as the ratio of falsely detected pixels to total detected pixels [6], as shown in Table 3.

The TDR of the three types of defect detection results achieved by the method in this paper is more than 90 percent, and the FDR can be controlled within an acceptable range. This shows that our method can detect crosstalk defects effectively and stably, and can achieve accurate detection of low-contrast defects.

### 4.4. Comparison of Different Methods

#### 4.4.1. Channel Selection Comparison

**Figure 7:**
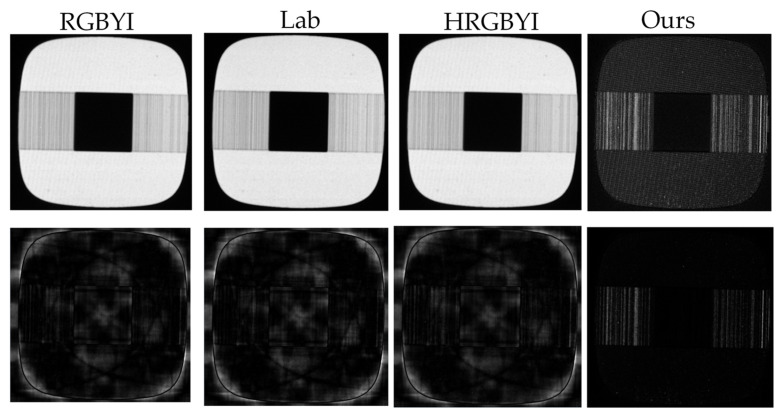
Combined feature saliency result graph: the first row is the quaternion modulo image, and the second row is the result saliency graph.

We compare the detection effects of the commonly used input feature combinations including Lab [20], RGBYI [29] and HRGBYI [33], which constitute the image quaternion matrix, with our proposed method, as shown in Figure 7.

It can be seen that the combination of Lab and RGBY cannot detect crosstalk defects, while the saliency map focuses on the edge parts. The detection results of HRGBYI, which is proposed to detect fabric defects, is also concentrated on the edge parts, keeping only a small amount of actual defect information. Compared with the above input feature combinations, which cannot effectively obtain the saliency map of defects, the combination of input features we proposed can effectively achieve the separation of defects and backgrounds.

#### 4.4.2. Algorithm Detection Effect Comparison

Our proposed method is compared with the current state-of-the-art defect detection methods to analyze crosstalk defect detection capabilities, including polynomial fitting [23] and discrete cosine fitting [22] based methods.

**Figure 8:**
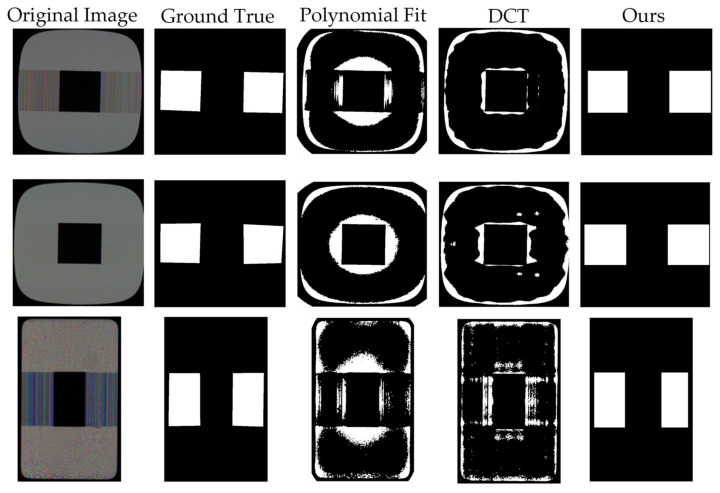
Crosstalk defect detection results from different methods.

As shown in Figure 8, the polynomial fitting method has a poor fitting ability for the cross-test picture, with the detection results concentrated in the edge part, which is seriously inconsistent with the actual defect position. The DCT method also has poor performance on the edge parts, and fails to overcome the special shape of the crosstalk pictures. It can be seen that the background reconstruction method has high requirements on the image and cannot have edge information. Our method can detect the defect areas more effectively, regardless of how strong or weak the contrast is, and succeed in overcoming the pollution caused by background noise.

## 5. Conclusions

We propose a crosstalk defect detection method based on salient color channel frequency-domain filtering. Firstly, the feature extraction and combination of images are analyzed and verified, and an effective feature extraction method for crosstalk defects is realized. For frequency domain filtering, we propose the GTB filtering method, which realizes the detection of low-contrast defects. We demonstrate the effectiveness of our method with detailed experiments and comparisons, and finally, show that our method can detect display crosstalk defects more accurately than the mainstream detection methods.

## Figures and Tables

**Table 1:** Signal-to-noise ratio and contrast of input images.

	Type 1	Type 2	Type 3
MSE	37.40	32.56	50.73
PSNR (dB)	32.40	33.00	31.07

**Table 2:** Background suppression and noise immunity comparison.

	1		2		3	
	SCRG	BSF	SCRG	BSF	SCRG	BSF
RGB	2.84	3.02	2.81	3.01	2.90	3.01
Lab	0.73	224.49	0.50	221.27	0.73	9200.90
Entropy H	1.45	40.86	1.58	35.83	1.55	20.00
HSV	2.89	548.11	3.10	568.41	1.89	639.49
RGBY	0.99	70.57	0.62	36.71	1.02	25.43

**Table 3:** Defect detection capabilities of our method.

	Type 1	Type 2	Type 3
TDR (%)	96.7	100	92.3
FDR (%)	7.6	11.8	4.5

## Data Availability

Not applicable.
